# *Meloidogyne marylandi* is Involved in, but not the Primary Cause of Creeping Bentgrass Decline of Putting Greens in Southern California

**DOI:** 10.2478/jofnem-2024-0046

**Published:** 2024-11-23

**Authors:** A. T. Ploeg, H. Witte, S. A. Subbotin, I. Tandingan De Ley, J. Smith Becker, J. O. Becker

**Affiliations:** Department of Nematology, University of California Riverside, 3401 Watkins Drive, Riverside, CA 92521; Plant Pest Diagnostics Center, California Department of Food and Agriculture, Sacramento, CA

**Keywords:** *Agrostis stolonifera*, creeping bentgrass, golf course, host range, *Meloidogyne marylandi*, nematode-degree days, morphology, molecular identification, root-knot nematodes, turfgrass

## Abstract

Root-knot nematodes were discovered in severely declining creeping bentgrass putting greens at a golf course in Indian Wells, Riverside County, California. The exhibited disease symptoms included chlorosis, stunted growth, and dieback. Based on morphological examination and measurements of J2 females and males, it was suggested that the causal pathogen was *Meloidogyne marylandi*. This identification was confirmed by analysis of the D2-D3 expansion segments of 28S rRNA and *COI* gene sequences. The host status of 28 plant species was evaluated in greenhouse trials. All tested monocots, except rye and *Allium* species, were found to be hosts, while no reproduction occurred on dicots. Temperature-tank experiments helped determine that the life cycle of *M. marylandi* was completed between 17–35 °C, with a base temperature of 8.3 °C and a required heat sum of 493 degree-days (DD). In greenhouse trials in pasteurized soil and near-ideal growing conditions, *M. marylandi* did not cause significant growth reduction of creeping bentgrass cv. Penn A-4, even at very high J2 inoculation densities. It is highly probable that other biotic and abiotic factors contributed to the observed putting green damage.

The Coachella Valley, an inland desert in Southern California, features 123 golf courses, comprising approximately 14 percent of California’s golf industry. It is an essential part of the economic vitality of the greater Palm Desert region, generating nearly $1.1 billion in overall economic activity and supporting more than 14,000 jobs (TE-Oxford Economics, 2015).

Among the many plant-parasitic nematode species associated with turfgrasses that cause severe damage to golf course greens in many parts of the world (Dong et al., 2022), root-knot nematodes (*Meloidogyne* spp.; RKN) are the most widespread plant-parasitic nematodes in California golf courses (Westerdahl et al., 2016). In a survey on the occurrence of RKN in California golf courses, *Meloidogyne naasi* was the most common species, but its distribution was limited to cooler or coastal areas (McClure et al., 2012). In contrast, *M. graminis* and *M. marylandi* were detected almost exclusively in the warm inland areas of Southern California (McClure et al., 2012). Separating these two species morphologically is difficult (Golden, 1989; McClure et al., 2012; Ye et al., 2015) and is currently done using molecular techniques (McClure et al., 2012; Ye et al., 2015). Another survey by the California Department of Food and Agriculture (CDFA) confirmed the presence of *M. marylandi* in golf courses in many southern California counties (CDFA Pest and Damage Records).

Two other plant-parasitic nematodes cause significant but localized problems in California turf: 1) The stem-gall nematode (*Anguina pacificae*) on annual bluegrass (*Poa anna*), which occurs in a few golf courses along a narrow strip along the Northern California coast (Cid del Prado Vera and Maggenti, 1984; McClure et al., 2008; Chitambar et al., 2018), and 2) The sting nematode (*Belonolaimus longicaudatus*) which was first detected in 1992 to 1993 in eight golf courses around Rancho Mirage, Coachella Valley, California, where it is a destructive, invasive pathogen in bermudagrass golf courses (Mundo-Ocampo et al., 1994). In California, this nematode has not spread beyond the original discovery sites (Chitambar et al., 2018) thanks to State and County regulatory restrictions.

The organophosphate nematicide fenamiphos was the primary turfgrass management tool against plant-parasitic nematodes for more than 30 years (Crow, 2014) until its registration was canceled in 2008 by the US EPA. In the fall of 2009, symptoms of decline in creeping bentgrass (*Agrostis stolonifera* L. ‘Penn A-4’) from 16-year-old putting greens were observed at a golf course in Indian Wells, CA. Areas with poor growth were first noticed in 2005, with the decline accelerating in the following years. Although slow decline of golf course putting greens is frequently observed in hot summer conditions (Huang and Gao, 2000; Xu and Huang, 2000), nematodes are generally overlooked as playing a potential role in this phenomenon. However, here we were made aware of this problem, and high numbers of RKN were isolated from root and soil samples collected from affected areas. The objectives of this study were to determine the role of RKN in the decline in this particular golf course and to study and document the pathogen’s biology, including its life cycle and the damage to creeping bentgrass.

## Materials and Methods

### Inspection of turf samples from declining golf greens

Turf samples were collected from four declining creeping bentgrass greens with sandy soil several hundred yards apart at a golf course in Indian Wells, Riverside County, California. From each green, five cores were taken with a soil sampling probe (2 cm diameter, Oakfield Apparatus Inc., Fond du Lac, WI) to approximately 10 cm depth, pooled, and stored in a cooler at approximately 15 °C until processing. Soil was washed from the root-thatch mat layer and roots were examined for the presence of galls. They were incubated in a mist chamber for nematode extraction (modified Seinhorst, 1950). Second-stage juveniles (J2) were counted after 5 days of incubation at 26 °C. Some roots were stained in acid fuchsin (Hooper, 1970) to visualize developing juveniles in the tissues. About 50 J2 were sent to the CDFA for molecular identification.

### Nematode isolation and culturing

One RKN egg mass from a creeping bentgrass root was used to inoculate creeping bentgrass cv. Penn A-4 that had been seeded in a 164 ml plastic tube (Ray Leach “Cone-tainers” Nursery, Canby, OR, USA) filled with pasteurized sand. This creeping bentgrass-RKN culture was maintained in a greenhouse at 25 ± 2 °C under ambient light. Eight weeks later, the creeping bentgrass roots were rinsed free of sand with a gentle stream of water and placed in a misting chamber for 2 weeks to obtain J2. This population was further reared in larger pots (800 cm^3^) filled with pasteurized sand and seeded with creeping bentgrass cv. Penn A-4, bermudagrass (*Cynodon dactylon* ‘Tifway’), rye (*Secale cereale* ‘Merced’), and barley (*Hordeum vulgare* ‘Morex’). Eight weeks later, J2 were extracted from the root systems as described above. Since many J2 were obtained from barley cv. Morex, this plant was used to further multiply and maintain the RKN population as the source of nematodes for morphological examination, molecular identification, and inoculum for experiments.

### Morphology and morphometrics

RKN females, J2, and males were obtained from infested barley cv. Morex roots. Thirteen females were excised from the roots, and perineal patterns were cut and transferred to glycerin on microscope slides for examination. Males (11) and J2 (25) obtained from roots after mist-chamber extraction were heat-killed, processed to glycerin, and mounted on microscope slides for measurement and examination (Seinhorst, 1962).

### Molecular identification

DNA was extracted from several J2 using proteinase K. DNA extraction and PCR protocols were conducted according to Subbotin (2021). Two primer sets were used for amplification of nematode genes: i) D2A (5′ - ACA AGT ACC GTG AGG GAA AGT TG - 3′) and D3B (5′ - TCG GAA GGA ACC AGC TAC TA - 3′) amplifying the D2-D3 expansion segments of 28S rRNA gene and ii) JB3 (5′ - TTT TTT GGG CAT CCT GAG GTT TAT - 3′) and JB5 (5′ - AGC ACC TAA ACT TAA AAC ATA ATG AAA ATG - 3′) primers for amplifying the partial *COI* gene. The successfully amplified fragments were purified and then sequenced by Azenta (MA, USA) with the primer pairs used in PCR. A Blastn search was used to compare the sequences obtained with those deposited in the GenBank. The new sequences were submitted to the GenBank database under accession numbers PP949975 (*COI* gene) and PP951498 (28S rRNA gene).

### Host range

The host status of 26 plant species (Table 1), comprising several graminaceous and dicotyledonous crop species, was evaluated in 2 separate greenhouse trials for this *Meloidogyne* species. Plants were seeded in 800 cm^3^ plastic pots containing pasteurized sandy soil. For each plant species, 3 pots were seeded with an amount of seed depending on the size of the mature plant. Two weeks after the emergence of the seedlings, the pots were inoculated with 3,500 RKN eggs from the previously described barley culture plants. After inoculation, the pots were randomized on a greenhouse bench, watered and fertilized through an automated drip irrigation system, and grown for another 6 weeks at 25 ± 2 °C. The roots were carefully rinsed off, blotted dry, and weighed. Eggs were extracted from the roots by shaking for 3 min in a 0.5% NaOCl solution (Hussey and Barker, 1973) and counted with a Hawksley counting chamber under a dissecting microscope at 40x magnification. The complete experiment was repeated once. The reproduction factor RF was calculated as Pf/Pi (number of eggs per root system at harvest/3,500).

**Table 1:** Average n = 6 reproduction factor (RF) final population/initial population of *Meloidogyne marylandi* on 28 crop cultivars grown in 1-liter pots 6 weeks after inoculation with 3,500 *M. marylandi* eggs per pot.

**Species**	**Common name**	**Cultivar**	**Reproduction factor^1^**
*Hordeum vulgare*	Barley	Morex	38.57 ± 12.65 a
*Avena sativa*	Oat	California Red	35.12 ± 13.79 a
*Zea mays*	Sweet Corn	Silver Queen	16.16 ± 6.25 a
*Triticum aestivum*	Wheat	Ojo Rojo	12.19 ± 2.33 a
*Agrostis stolonifera*	Creeping Bentgrass	Penn A-4	6.46 ± 4.12 bcd
*Cynodon dactylon*	Bermudagrass	Southern Starr	4.97 ± 2.02 ab
*Cynodon dactylon*	Bermudagrass	Numex Sahara	4.61 ± 1.11 ab
*Lolium perenne*	Perennial Ryegrass	Zoom	4.09 ± 1.28 ab
*Festuca ovina glauca*	Blue Fescue	Elijah Blue	3.77 ± 0.87 ab
*Poa annua*	Annual Bluegrass	Two Putt	3.11 ± 0.79 abc
*Cynodon dactylon*	Bermudagrass	Sundevil II	2.00 ± 0.79 bcde
*Festuca rubra*	Red Fescue	Commutata J5	1.71 ± 0.61 bcdef
*Poa pratensis*	Kentucky Bluegrass	4-Season	0.71 ± 0.27 efg
*Festuca arundinacea*	Tall Fescue	Watchdog	0.51 ± 0.21 fg
*Allium porrum*	Leek	American Flag	0.23 ± 0.10 defg
*Agrostis capillaris*	Colonial Bentgrass	Jorvik	0.11 ± 0.04 efg
*Allium schoenoprasum*	Chives	Garlic	0.08 ± 0.02 fg
*Allium cepa*	Onion	Yellow Granex	0.06 ± 0.04 gh
*Arachis hypogaea*	Peanut	Florrunner	0.00 ± 0.00 h
*Capsicum annuum*	Pepper	California Wonder	0.00 ± 0.00 h
*Secale cereale*	Rye	Merced	0.00 ± 0.00 h
*Beta vulgaris*	Sugarbeet	5 GK 1122	0.00 ± 0.00 h
*Citrullus lanatus*	Watermelon	Charleston Grey	0.00 ± 0.00 h
*Brassica oleracea var. italica*	Broccoli	Liberty	0.00 ± 0.00 h
*Brassica oleracea*	Cabbage	Copenhagen Market	0.00 ± 0.00 h
*Cucumis melo*	Cantaloupe	Durango	0.00 ± 0.00 h
*Gossypium hirsutum*	Cotton	GC 510	0.00 ± 0.00 h
*Cucumis sativus*	Cucumber	Imanol	0.00 ± 0.00 h
*P*-value crop cultivar effect: < 0.0001			

1 Values shown are the mean of 6 replicates n = 6±SE. Statistical analysis was done using the Kruskall-Wallis test to test the significance of the crop cultivar on Log_10_[eggs per root system +1]-transformed data. Values in a column followed by different letters are significantly different *P* ≤ 0.05 according to the Bonferroni test.

### Effect of soil temperature on the duration of the life cycle

Soil temperature is the main abiotic factor determining the rate of development of RKN (Trudgill, 1995; Giné et al., 2021). Consequently, its effect was the focus of this part of the study. The methodology was similar to the one used by Lahtinen et al. (1988). One-liter plastic pots were filled with 800 cm^3^ steam-pasteurized river bottom sand (90% sand, 1% silt, 8% clay, 0.2% OM, pH 7.5), seeded with barley cv. Morex (2 seeds/pot) and placed on a greenhouse bench for three weeks to germinate. The pots were then placed in water baths, with the soil level inside the pots lower than the water level outside. The tops of the pots were insulated with polystyrene disks with holes for the barley plants. The pots were then transferred to water baths set at 17, 20, 25, 30, and 35 °C (five pots for each temperature). A temperature reader (HOBO, Spectrum Technologies, Inc., Plainfield, IL, USA) set to record the temperature at one-hour intervals was inserted into one pot in each water bath.

Two days later, 2 ml of a J2 suspension, containing approximately 640 J2 (experiment 1) or 2,000 J2 (experiments 2–4), obtained from infested barley cv. Morex roots, were pipetted into 4 shallow holes close to the stems of the barley plants. Plants were grown and fertilized once with 5 g of controlled-release fertilizer (Osmocote 17-6-10; Scotts-Sierra Horticultural Products Co, Marysville, OH) until 20 days after nematode inoculation. Plants were carefully removed from the pots, and the roots were washed free of soil with a gentle stream of water. They were transferred to new, similar pots with five 3-mm-diam. holes drilled in the bottom of each pot. The pots were carefully three-quarters filled with washed small (ca. 5-mm-diam.) stones, placed inside similar pots without holes in the bottom, and returned to the water baths. To collect newly-hatched J2, 300 ml water was added to the inside pot every day and allowed to percolate through the pot. The J2 in the percolate were collected on two 500-mesh (25-micron-pore-size) sieves and counted under a dissecting microscope at 40× magnification.

This entire experiment was done four times at four-week intervals. After the first experiment, the transfer of the barley plants to the pots with stones was not done on the same day for each temperature, but was based on results obtained from the first experiment – i.e., four days prior to the expected first appearance of J2. The second experiment was an exact repeat of the first experiment, but due to a technical problem with the 17 °C water bath, this treatment was lost in the second experiment. The third experiment included 17 °C and temperatures between 25 °C and 33 °C with 2 °C intervals (17, 25, 27, 29, 31, and 33 °C). The fourth experiment was basically a repeat of the third experiment, but with only the 27, 29, and 31 °C treatments.

To qualify as the “first day of life cycle completion”, J2 had to be recovered from at least two out of five replicates, and the number of recovered J2 on each of the two days following the initial recovery had to be higher than on the previous day. In each experiment, J2 were collected for at least one week following the initial recovery.

To estimate the base temperature and degree-days required for life cycle completion, a regression analysis was done according to:

R=1/S×Ts−Tb/S

With R = reproductive rate [1/ (days to life cycle completion)], S = required heat sum (DD = degree-days), Ts = average soil temperature (°C), and Tb = base temperature (°C).

### Damage potential in creeping bentgrass

Creeping bentgrass cv. Penn A-4 was grown from seed in 164 cm^3^ plastic cones (Ray Leach “Cone-tainer” Nursery, Canby, OR) filled with pasteurized plaster sand. The cones were inoculated at seeding with 0, 100, 1,000, 5,000, or 10,000 *M. marylandi* J2 and placed in a greenhouse at 25 ± 2 °C under ambient light. The cones were fertilized once every four weeks by drenching with a solution of 5g/L Miracle-Grow (The Scotts Company LLC, Marysville, OH, USA). The trial design was a randomized complete block with 6 replications per treatment, and the trial was repeated once. The grass in each cone-tainer was cut back to approximately 1 cm every 2 weeks, and the fresh weights of the clippings were determined. Nematode damage was expected to reduce the fresh weight of the clippings in the inoculated treatments compared to non-inoculated controls. Both trials were terminated 180 days after seeding. At this time, the roots were carefully rinsed and placed in a mist chamber for 14 days at 26 °C to extract J2.

### Statistical analysis

A Shapiro test (at *P* = 0.05) was applied to the collected data to test if the data conformed to a normal distribution. If data were normally distributed, ANOVA tests were done to determine the significance of the treatment effects, the repeated experiments, and their interactive effects. Post-hoc separation of means was done using Fishers’s Least Significant Difference (LSD) test (at *P* = 0.05). If the data were not normally distributed, a non-parametric Kruskall-Wallis test was used to determine the significance of treatment effects, and post-hoc separation of means was done using a Bonferroni test (at *P* = 0.05). R-language (R Core Team, 2013) was used for statistical analysis.

## Results

### Inspection of turf samples from declining golf greens

Above-ground symptoms on creeping bentgrass greens were typical for nutritional problems, with chlorosis, stunted growth, and dieback. Mist chamber extraction of soil and root samples from affected creeping bentgrass areas yielded several hundred RKN J2 per 100 cm^3^ soil. The roots showed galls typical of root-knot nematode infestation and exhibited light-to-dark-brown discoloration. Staining the roots with acid fuchsin revealed numerous advanced juvenile stages and females of root-knot nematodes.

### Morphology and morphometrics

**Figure 1:**
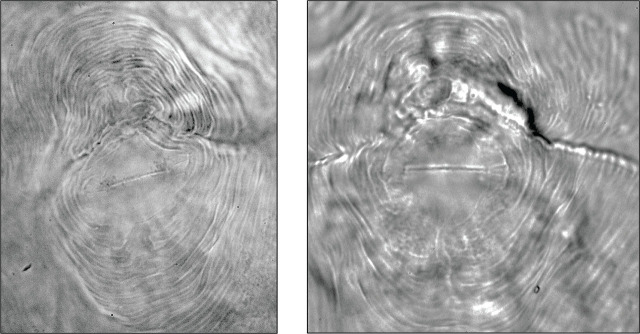
Example of two perineal patterns of a *Meloidogyne marylandi* population isolated from creeping bentgrass in Indian Wells, California.

Males were fairly common in the population that was multiplied on barley cv. Morex. The J2 (n = 25) mean body length was 396.7 ± 3.7 (352.8 – 438.1) μm, and the mean body width was 16.1 ± 0.12 (15.1 – 16.9) μm. Male (n = 11) mean body length was 1127.6 ± 34.9 (864 – 1273) μm. Perineal patterns (n = 13) were rounded-to-ovoid with coarse striae (Figure 1) and conformed to the description of *Meloidogyne marylandi* Jepson and Golden, 1987.

### Molecular identification studies

The sequence of the D2-D3 expansion segments of the 28S rRNA gene showed 100% similarity with that of *M. marylandi* from bermudagrass, USA (KP901066). The *COI* gene sequences showed the highest similarity (98.8%) with *M. marylandi* from Japan (ON453615 and ON171452).

### Host range

**Figure 2:**
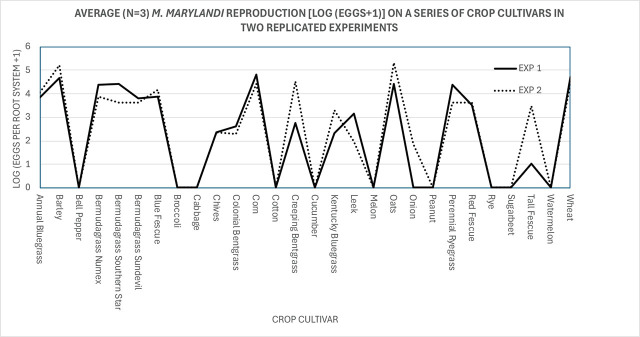
Average (n = 3) egg production [Log_10_(eggs per root system) + 1] of *Meloidogyne marylandi* on 28 crop cultivars in two replicated experiments, 6 weeks after inoculation with 3,500 eggs.

Statistical analysis was applied to Log_10_[(eggs per root system) +1]-transformed data. ANOVA analysis showed that the experiment (Exp1, Exp2), the crop cultivar, and their interaction were all significant at the 95% confidence level. However, examination of the interaction plot (Figure 2) showed that the results were very similar between the two replicated experiments, with a few exceptions: reproduction on creeping bentgrass, tall fescue, and onion was considerably lower in experiment 1 than in experiment 2. Despite these minor differences, data between the two replicated experiments were combined and analyzed as the average of six replicates. Because the non-transformed and the Log_10_[eggs per root system +1]-transformed data did not fulfill the requirements of a normal distribution (Shapiro test; *P* ≤ 0.05), the non-parametric Kruskall-Wallis test was used to test the crop-cultivar effect on the nematode reproduction. The Bonferroni test (at *P* = 0.05) was used to separate the means. The reproductive factor (RF = Pf / Pi) for host plants was calculated by dividing the number of eggs extracted from the root systems per pot by the number of eggs used as inoculum.

The host status of the different crop cultivars was evaluated based on the reproduction factor (RF) of *M. marylandi* on those crop cultivars (Table 1). An RF ≥ 10 was considered an excellent host, 10 < RF < 1 a good host, RF ≈ 1 a maintenance host, and RF < 1 a poor to non-host (Ferris et al., 1993). The grain crops barley, oats, corn, and wheat were excellent hosts for *M. marylandi* and were the only crops with RF-values > 10. Most grasses (except Kentucky bluegrass, tall fescue, and colonial bentgrass) were good hosts, allowing the population to increase. All dicotyledonous plants tested were poor or non-hosts (Table 1). Surprisingly, rye, a crop closely related to wheat and barley (Clifton and Keogh, 2016), was not a host for *M. marylandi* in these tests.

### Effect of soil temperature on the duration of the life cycle

The life cycle was completed at each of the nine tested soil temperatures, and the results were generally very similar between the experiments. One exception was the result obtained at 31 °C; in experiment 3, it took 24 days for life cycle completion, whereas in experiment 4, it took 32 days (see Table 2). At the lowest temperature tested (17 °C), the number of days required for life cycle completion was the longest (55 and 59 days in exp. 1 and exp. 3; Table 2). Life cycle completion was fastest at 29 °C (23 and 24 days in exp. 3 and exp. 4; Table 2). At higher soil temperatures, the number of days required to complete the life cycle increased again, and it took about 6 days more at 35 °C than at 29 °C (Table 2).

**Table 2:** Effect of soil temperature (°C) on number of days to complete the life cycle of a *M. marylandi* population.

**Soil temperature**	**Experiment**			
	1	2	3	4
17	55		59	
20	41	41		
25	29	30	30	
27			27	26
29			23	24
30	24	25		
31			24	32
33			25	
35	29	29		

In general, the effects of soil temperature on nematode reproduction during the first five days after life-cycle completion corresponded with the temperature effects on the life-cycle duration. At the highest (35 °C) and lowest (17 °C) temperatures tested, nematode reproduction was low, with only about 3% and 10% of the number of nematodes recovered relative to the number inoculated. In the temperature range from 27 to 30 °C (around the optimum temperature for life cycle completion), the reproduction factor RF (nematode recovered/nematodes inoculated) was greater than 1 in each experiment (Table 3).

**Table 3:** Average n=5 reproduction factor RF (number of J2 recovered/number of J2 inoculated) of a *M. marylandi* population during the first five days after life cycle completion at different soil temperatures (°C).

**Soil temperature**	**Experiment**			
	1	2	3	4
17	0.06		0.13	
20	0.22	0.93		
25	2.06	0.93	5.20	
27			14.12	6.85
29			2.71	3.00
30	1.66	2.94		
31			1.60	0.11
33			0.28	
35	0.04	0.01		

**Figure 3:**
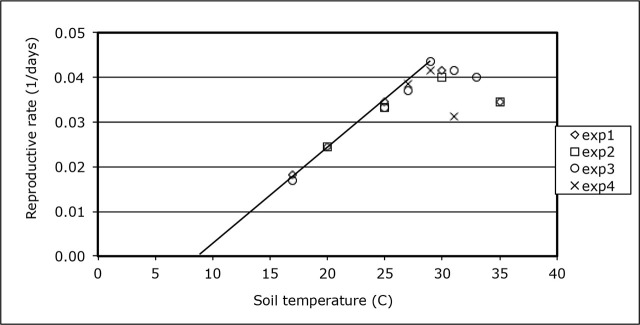
Effect of soil temperature on the reproductive rate R (days-1) of *Meloidogyne marylandi* in four experiments. Line represents regression analysis : R = 0.0020 × Ts-0.0168 for Ts = 17 – 29 °C, according to R = 1/S × Ts - Tb/S, with S = required heat sum (degree-days), Ts = soil temperature (°C), and Tb = base temperature (°C) for reproduction. Correlation coefficient *R^2^* = 0.998.

Plotting the reproductive rate (1/day) against soil temperature showed that the reproductive rate at 31 °C from experiment 4 did not correspond with the rest of the data (Figure 3). This data point was omitted in the regression analysis. Figure 3 also shows that the reproductive rate starts to decrease at 30 °C and higher soil temperatures. A regression line was thus fitted through the data points over the 17 to 29 °C range. The resulting regression function

R=0.0020T−0.0168

explained over 99% of the observed variation (correlation coefficient *R^2^* = 0.998). According to this function, *M. marylandi* has a required heat sum of 493 degree-days and a base temperature of 8.3 °C, below which reproduction will not occur.

### Damage potential in creeping bentgrass

The effects of the two trials – the different inoculation levels and their interactive effect on the total cumulative weight of the grass clippings, the root weight at harvest, and J2 levels at harvest – were statistically analyzed. Data on the total fresh weight of the clippings, root weight, and Log_10_[x+1]-transformed J2 data satisfied normal distribution requirements (Shapiro test; *P* > 0.05).

**Figure 4:**
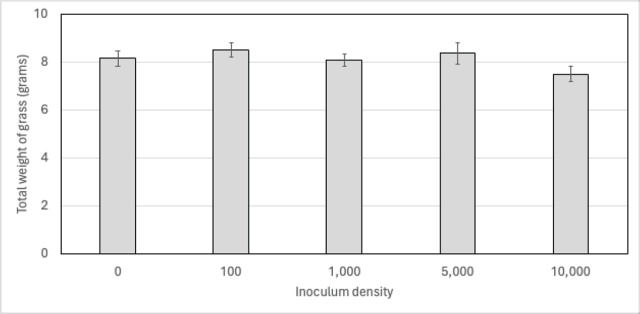
Average (n = 12) total fresh weight of creeping bentgrass (*Agrostis stolonifera*) cuttings over a 180-day period under a range of *Meloidogyne marylandi* second-stage juvenile inoculum densities. Bentgrass was grown in 164 cm^3^ plastic cones in a greenhouse and cut every two weeks.

ANOVA analysis showed that inoculum density did not significantly affect the total weight of the grass clippings (*P* > 0.05). The total weight of the grass clippings was significantly higher in the first than in the second trial (8.54 and 7.71 gram, respectively). The interaction between the experiment and inoculum density was not significant (*P* > 0.05). Therefore, the weights of the grass clippings under the different inoculum levels are shown as the average of the two replicated trials (n = 12; Figure 4). The average root weight at harvest was not significantly affected by the experiment, the inoculum density, or their interaction (*P* > 0.05). No J2 were found in the roots from the non-inoculated treatments. In the inoculated treatments, the average number of J2 per root system at harvest (overall: 2,264) was not significantly different in the two replicated experiments or among the different inoculum levels (*P* > 0.05). The interactive effect between the experiment and inoculum level was also not significant (*P* > 0.05).

## Discussion

Based on the results from morphological examination, measurements, and molecular identification, we concluded that the RKN species associated with stunting and yellowing of the creeping bentgrass greens at an Indian Wells, California golf course was *M. marylandi*. Although some studies (Oka et al., 2007; Salazar et al., 2013; Jagdale et al., 2019) reported slightly larger body length measurements for *M. marylandi* J2, our measurements are very similar to those reported from field populations in Oklahoma, Maryland, and Florida (Golden, 1989; Sekora et al., 2012; Walker, 2014). Also, whereas in this CA population males were relatively common, others reported males as absent or rare (Golden, 1989; Oka et al., 2004; Salazar et al., 2013).

The shortest time for life cycle completion was estimated to occur at 29 °C. At this temperature, the life cycle was completed in as little as 24 days. This is very similar to the reported optimum temperature of 28 °C for the grass root-knot nematode *M. graminis,* although the time needed to complete the life cycle was considerably longer, at 36 days (Grisham et al., 1974).

Most grasses and grain crops were excellent or good hosts for this California *M. marylandi* population. In contrast, all dicotyledonous plants tested were non-hosts, as no eggs were recovered from their roots. Others also have reported grasses to be good hosts for this nematode (Jepson and Golden, 1987; Kimmons et al.,1990; Araki, 1992; Oka et al., 2003; 2004; Faske and Starr, 2009), and dicotyledonous plants to be non-hosts (Oka et al., 2003; Faske and Starr, 2009). Results on some grain crops were more variable. The susceptibility of wheat and barley to *M. marylandi* has been reported previously (Oka et al., 2003; Faske and Starr, 2009). In our study, oats and corn were also excellent hosts, but others reported these crops as poor or non-hosts (Oka et al., 2003; Faske and Starr, 2009). The host range for *M. marylandi* from our study was very similar to those reported for other California grass RKN, such as *M. graminis* and *M. naasi* (Ferris, 2024). In this study, rye and sugarbeet were non-hosts for *M. marylandi* but were reported to be hosts for *M. naasi* (Kort, 1972; Michell et al., 1973).

Increasing inoculum densities of *M. marylandi* did not result in differences in the growth of creeping bentgrass cv. Penn A-4, indicating that creeping bentgrass is a tolerant host under nearly ideal growing conditions in the greenhouse and without additional biological or physical stress. Also, at the harvest of the trial – 180 days after inoculation – the number of J2 recovered from the creeping bentgrass roots were similar among all the inoculated treatments (average: 2,264 J2 per root system). This suggests that the creeping bentgrass root system could not sustain higher populations of this nematode, perhaps due to being limited by the number of available feeding sites.

The absence of observed damage stands in stark contrast to the severe decline observed in the creeping bentgrass greens at the golf club in Indian Wells, CA. Therefore, factors other than the nematodes must have played an important role in causing the observed damage. Creeping bentgrass is a cool-season grass with optimal temperatures for shoot growth ranging from 18 to 24 °C and from 10 to 18 °C for root growth (Beard, 1973). At Indian Wells, CA, however, day temperatures during July and August are consistently over 38 °C, which causes heat stress to the creeping bentgrass and can result in significant decline, particularly when it is mowed short and frequently (Huang and Gao, 2000; Xu and Huang, 2000; Liu and Huang, 2002; Pote et al., 2006). Additional biological stress factors, such as RKN interactions with secondary pathogens like root-invading microorganisms (Mayol and Bergeson, 1970; Back et al., 2002), may have further increased the susceptibility of the creeping bentgrass to decline during unfavorable growing conditions.
